# Psychosocial beliefs of health providers' intention and behavior of offering HIV testing and counseling services: Estimating their relevance for intervention

**DOI:** 10.3389/fpubh.2022.796035

**Published:** 2022-10-19

**Authors:** Almutaz Mohammed Idris, Rik Crutzen, Hubertus W. Van Den Borne

**Affiliations:** ^1^College of Applied Medical Sciences, Buraydah Colleges, Buraydah, Saudi Arabia; ^2^Department of Health Promotion, Maastricht University/CAPHRI, Maastricht, Netherlands

**Keywords:** beliefs, HIV testing, Confidence Interval Based Estimation of Relevance, healthcare providers, intention, behavior

## Abstract

**Background:**

Diagnosis of people with HIV is vital in achieving the 95-95-95 global targets. The proportion of people with HIV who know they have HIV in Sudan is low. Promoting engagement of healthcare providers (HCPs) in offering HIV Testing and Counseling (HTC) services would improve the percentage of diagnosed people with HIV in the country. This study aims to assess the psychosocial beliefs associated with HCPs' intention and behavior to offer HTC services and their relevance for intervention.

**Methods:**

This institutional cross-sectional study was conducted in Kassala State, from July 2019 to February 2020, among 438 healthcare providers from public health facilities. A self-administered questionnaire was used to assess behavior and intention to offer HTC services and related behavioral, normative, and control beliefs. Data were analyzed using R software. Confidence Interval Based Estimation of Relevance (CIBER) was used to estimate the relevance of the beliefs to interventions.

**Results:**

The CIBER analysis showed that the belief “It causes many worries for patients if I offer or counsel them about HIV test” was negatively associated with HCPs' intention and behavior to offer HTC services and a highly relevant belief for intervention. The belief “My manager thinks I should offer or counsel patients about HIV test” was positively associated with the behavior and intention to offer HTC services and was a relatively highly relevant belief. The control belief “Patients are at low risk of HIV and do not need offering or counseling about HIV test” was negatively associated with HCPs' intention and behavior and was relevant for intervention. The control belief “If I offered or counseled patients about HIV test, I would spend more time with them” was negatively associated with the intention and behavior of HCPs toward HTC services, with high relevance to target with intervention. The belief “My colleagues think I should offer or counsel patients about HIV test” was weakly associated with behavior and intention, and it is a low relevant belief for intervention.

**Conclusion:**

Different psychosocial beliefs among healthcare providers can influence their intention and behavior to offer HTC services to patients. More relevant beliefs are required to be targeted with interventions to promote the intention and behavior of providing HTC services among health care providers.

## Introduction

In 2014, The Joint United Nations Programme on HIV/AIDS (UNAIDS) set 95-95-95 targets to drive progress toward ending the HIV epidemic (1). These targets aim to diagnose 95% of people with HIV, 95% of those diagnosed to receive antiretroviral therapy, and 95% of people with HIV on treatment be virally suppressed by 2030. Performance on these targets varies between countries. In 2016, a systematic analysis of HIV treatment cascades in 69 countries showed that the percentage of people with HIV who knew their HIV status ranged from 87 to 11%, these on treatment ranged from 71 to 3% and 68 to 7% of people with HIV on treatment were virally suppressed (2). In Sudan in 2019, of the estimated people with HIV, only 27% knew their HIV status, and 56% were on treatment (3). This low percentage of diagnosed people with HIV in Sudan can be attributed to the low offer, demand, and uptake of HIV testing by the people with HIV.

To improve HIV testing rates, diagnose people with HIV, and progress toward the first 95% global target, it is essential to enhance the engagement of HCPs in offering HIV Testing and Counseling (HTC) services. Provider Initiated Testing and Counseling (PITC) is one of the strategies used to offer and create demand for HIV testing in health care settings and link those with HIV-positive results to treatment and care (4–6).

This approach differs from client-initiated HIV testing (voluntary counseling and testing), which links the offering of HIV tests to patient requests. Previous studies showed that PITC has helped increase the number of people tested for HIV in different health settings, including antenatal, tuberculosis, and outpatients clinics (7–9).

Sudan Ministry of Health adopted the PITC approach in 2009 in selected health facilities (10). Currently, it is provided in antenatal care, Tuberculosis (TB), and sexually transmitted infection (STI) facilities (11). Although the PITC has successfully encouraged people to take the HIV test, the testing rates are still below the national targets. Evidence showed that in Sudan, patients are more likely to accept HIV tests when offered by a health care provider. A study conducted among pregnant women in Sudan to measure their perception of HIV testing suggests that HIV tests can be accepted when offered by a doctor (12). This indicates that better encouragement of HCPs to offer HIV tests to their patients can increase the number of people who take the test in Sudan.

Many factors can influence the engagement of HCPs in offering HTC behaviors. Among these factors are inadequate private space for counseling, lack of time, workload, lack of guidelines (13–15) as well as social-cognitive influences (16, 17), e.g., intention, attitude, subjective norms, perceived behavior control and beliefs (18–21). Social cognitive models can help understand different factors that shape people's intentions and behaviors (22), including the behaviors of HCPs (23). The Reason Action Approach (RAA) is a social-cognitive model that proposes that individuals' behavior is directly influenced by the intention to perform the behavior (24). The intention is a product of attitude, subjective norms, and perceived behavioral control, which are formed by persons' behavioral, normative, and control beliefs regarding performing a behavior, respectively (21, 25, 26). Therefore, the psychosocial beliefs that individuals' have about behavior are indirectly influenced a person's intentions to engage and perform that behavior.

No previous study assessed the influence of psychosocial beliefs on HCPs' behavior and intention to offer HTC services and their relevance to interventions in Sudan. This study used the RAA to assess the psychosocial beliefs associated with HCPs' intention and behavior to offer HTC services and their relevance to be targeted with behavior change interventions to enhance HTC services provision intention and behavior among HCPs in Kassala state Sudan.

## Materials and methods

The study methods were reported in line with the STROBE Statement of cross-sectional studies (27).

### The study design and settings

An institutional-based cross-sectional study was conducted among HCPs in public health facilities in Kassala state from July 2019 to February 2020. Kassala state is one of eighteen states in Sudan situated in the country's eastern, a region with an estimated HIV prevalence of 4.18% (28), which is high compared to the national HIV prevalence of 0.2% (29). The state is divided into eleven localities and borders Ethiopia and Eretria, high HIV epidemic countries. The estimated population was 2.9 million in 2019, as projected from the 2008 population census report. According to the Kassala State Ministry of Health reports, the state has 19 public health hospitals (3 tertiary and 16 secondary hospitals) and over a hundred health centers (primary level) during the study period.

Ethical approval for the study was obtained from the Kassala State Ministry of Health Research Ethical Committee. Permissions were gained from the administrative authority of selected facilities.

### Participants and sampling

Participants were eligible to participate if they were doctors, nurses, and psychologists who worked in a public health facility, directly interacted with patients in their daily practice and had worked for at least 2 years. A sample of 438 participants was estimated based on the sample size estimation for correlation with 95% confidence intervals (CIs), width = 0.5, and correlation = 0.10 (30). Nineteen health facilities were selected randomly, including two tertiary, five secondary hospitals, and twelve health centers. Two health centers were selected from the Kassala locality (the most populated) and one health center from ten localities. Then a systematic sampling method was employed to recruit the participants at the selected health facilities. The first participant was chosen randomly then a fixed interval was applied. The next one replaced a respondent who refused to participate in the study.

### Data collection

Data was collected using a self-administered questionnaire. The questionnaire was pre-tested before data collection started. It was translated from English to Arabic (the official language in Sudan) and then translated back to English by language experts. The study's purpose and objectives were explained to participants, and they assured about the confidentiality of their responses. The respondents were then asked to complete the questionnaire in a private room within the health facility outside their working hours to avoid disrupting patient care. All participants provided consent before being included in the study.

### Variables and measurements

The study's outcome variables were HCPs' intention and behavior to offer HTC services. The explanatory variables were behavioral, normative, and control beliefs regarding offering HTC services.

A self-administered questionnaire was used to gather participants' socioeconomic and social-cognitive variables-related data. The socioeconomic variables included age, gender, academic degree, year of experience, and field of study. Questions for social cognitive variables were developed based on the previous studies' results (18, 22) and elicitation study findings (24). The elicitation study is conducted on a small group of participants from the study population to determine their most common salient beliefs (behavioral, normative, control beliefs) regarding the behavior under the study (offer HTC services behavior in this study). The more frequent beliefs reported by the participants regarding offering HTC services were included in the final questionnaire.

The behavior of offering HITC services was measured by one item: “I offered or counseled patients on HIV tests in the last 3 months.” Participants responded with “Yes” or “No.”

Intentions to offer HTC services were assessed by three items: “I intend,” “I expect,” and “I want” to offer or counsel patients about HIV tests in the next 3 months. Responses ranged from unlikely (+1) to likely (+7). A high score suggests a more positive intention to offer HTC services.

Behavioral beliefs were assessed with four statements: “If I offer or counsel patients about HIV test, I feel that I am doing something positive,” and “It causes many worries for patients if I offer or counsel them about HIV test.” “If I offered or counseled patient to get HIV test, I detect HIV cases at an early stage,” and “If I offer or counsel patients about HIV test, I would feel discomforted.” Answers ranged from unlikely (+1) to likely (+7).

Normative beliefs were assessed using three items. The items were “My manager thinks I should offer or counsel patients about HIV test,” “Patients think that I should offer or counsel them about HIV test,” and “My colleagues think I should offer or counsel patients about HIV test.” Answers were reported on a seven-point Likert scale [disagree (+1) and agree (+7)].

The control beliefs were evaluated by using four items which were answered on a seven-point Likert scale [with Unlikely (1) and Likely (7)]. They were “Patients are at low risk of HIV and do not need offering or counseling about HIV test” and “If I am trained, I would feel confident to offer or counsel patients about HIV test.” “If I offered or counseled patients about HIV test, I would spend more time with them,” and “Having HIV test guidelines would make it easier for me to offer or counsel patients about HIV test.”

### Data analysis

R software (31) was used for data analysis. The descriptive statistics were computed to describe the participants' characteristics, including proportion, mean, standard deviation, and range.

Confidence Interval Based Estimation of Relevance (CIBER) approach was used to assess the relevance of social-cognitive beliefs for interventions to promote the intention and behavior of offering HTC services among HCPs (32). The CIBER visualizes different information about the data in a plot with two panels (right-side and left-side) with colored diamonds. The left-side panel's diamonds show the means of sub-determinants (the social cognitive beliefs in this study) with 99% confidence intervals. The diamonds in the right-side panel view the association (i.e., correlation) of the sub-determinant with the outcome variables (intention and behavior to offer HTC services in this study) with 95% confidence intervals. The colors of the diamond provide extra information. For diamonds in the left-side panel, the redder indicates a low mean, the blue indicates a middle mean, and the greener indicates a high mean. In the right-side panel, the color of the diamond provides additional information on the association's strength and direction: greener suggests a strong positive association, redder indicates a strong negative association, and grayer indicates a weak association. The questions and anchors for each belief are presented on the side of the left-hand panel. The CIBER plot provides the explained variance of all explanatory variables in the outcome variables at the top of the plot.

## Results

### Descriptive statistics

Table 1 presents the socioeconomic characteristics of the study participants.

**Table 1:** Participants descriptive statistics (N = 438).

**Variables**	***N*** **(%)**	**M**	**SD**	**Range**
**Age in years**		34.11	7.46	26–59
**Gender**				
Female	273 (62.4%)			
Male	165 (37.6%)			
**Highest degree earned**				
Doctorate	67 (15.3%)			
Masters	152 (34.7%)			
Diploma	152 (23.1%)			
Bachelors	118 (26.9%)			
**Years of experience**		6.78	4.51	1–19
**Field of study**				
Medicine	216 (49.3%)			
Nurse	143 (32.6%)			
Psychologist	79 (18.1%)			

### CIBER analysis

**Figure 1:**
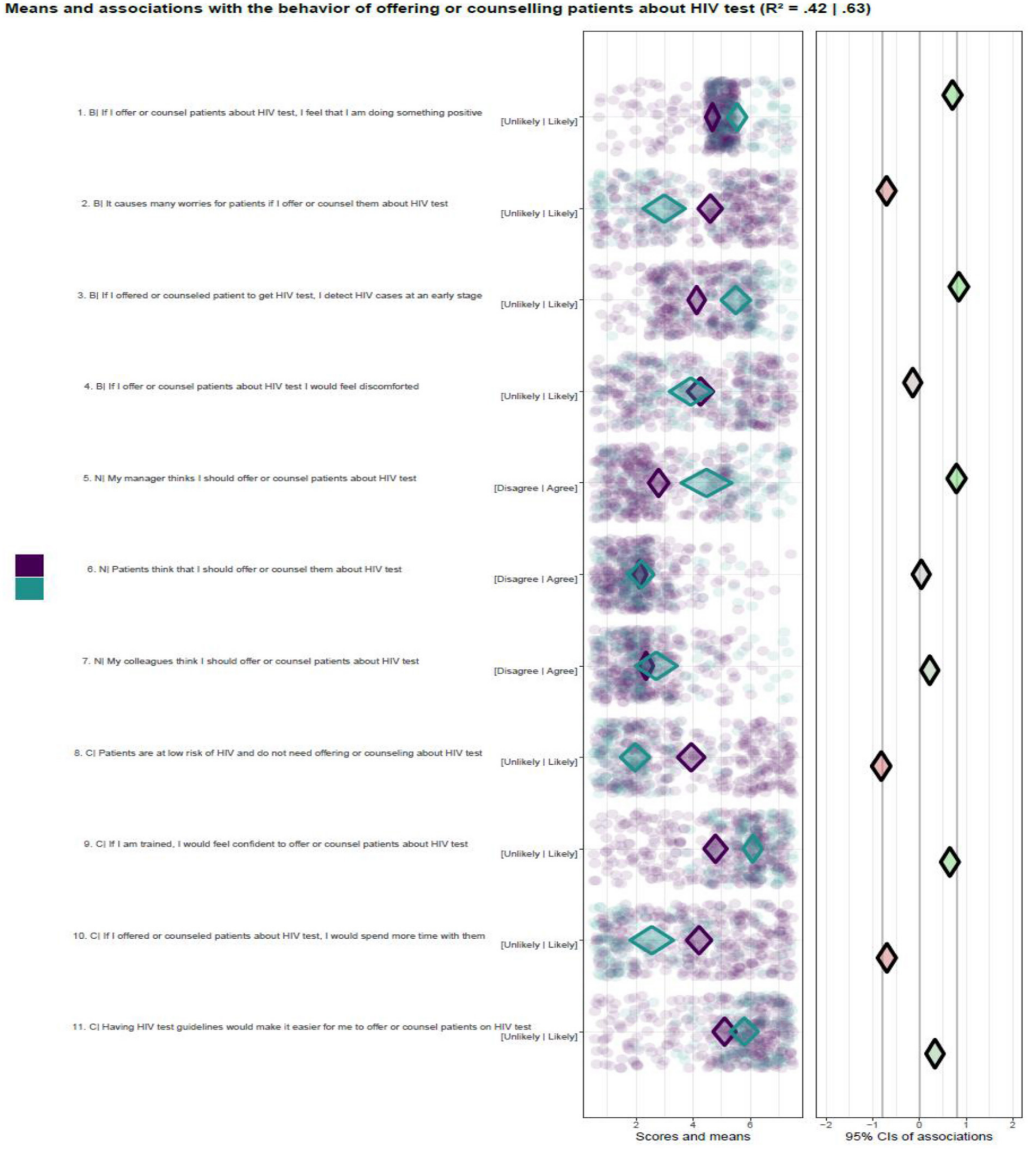
Psychosocial beliefs and Behavior of offering or counseling about HIV test. Purple color indicates means score for not performing the behavior, B, behavioral belief; N, normative beliefs; C, control beliefs.

Figure 1 shows the CIBER analysis output for the beliefs and behavior. The left panel has two colored diamonds, green for participants that reported “yes” on the behavior and purple-colored for those who said “no.” For all scales, most beliefs' mean scores of participants who did not perform the behavior with 99% CIs were above the middle of the scale. The mean scores for those who reported doing the behavior were below or around the middle of the scale, with the belief “If I am trained, I feel confident to offer or counsel patients about HIV test” as the higher mean score.

The participants' beliefs that managers think they should offer or counsel patients about HIV tests were positively associated with behavior. It can also be seen as a relevant belief for intervention because its scores indicate that more than half of the participants were convinced and performed the behavior.

The beliefs “It causes many worries for patients if I offer or counsel them about HIV test,” and “If I offered or counseled patients about HIV test, I would spend more time with them” were negatively associated with behavior. Also, the respondents' belief that “Patients are at low risk of HIV and do not need offering or counseling on HIV test” was strongly negatively associated with the behavior. The relevance of these beliefs can be viewed as relatively high because of association and below middle-scale scores for participants who performed the behavior. This indicates that a large proportion of the participants who do not offer HTC services had that belief.

**Figure 2:**
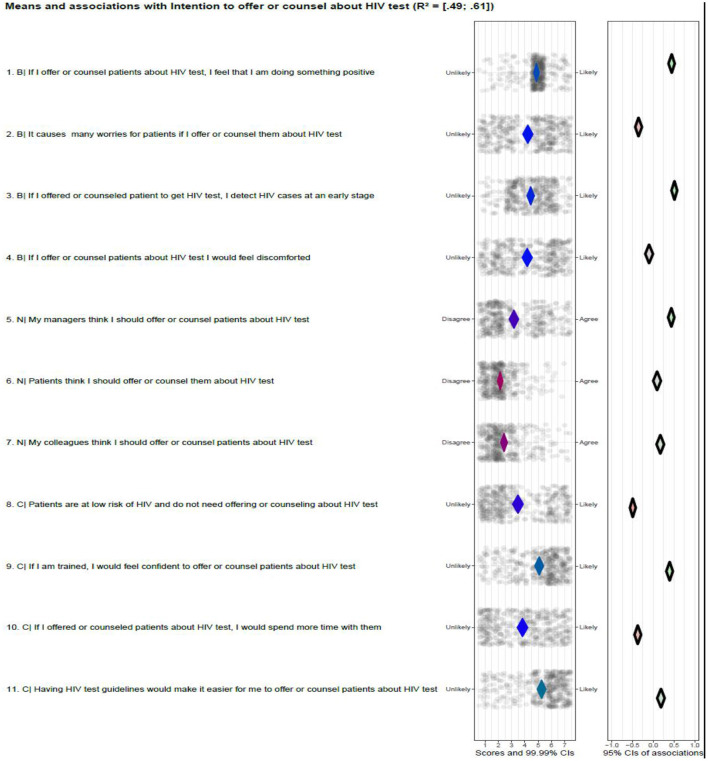
Psychosocial beliefs and intention to offer or counseling about HIV test. B, behavioral belief; N, normative beliefs; C, control beliefs.

The intention's explained variance (*R*^2^) based on all beliefs ranged from 0.49 to 0.61 (Figure 2). The beliefs” If I offer or counsel patients on HIV test, I feel that I am doing something positive” and “My manager thinks that I should offer or counsel patients about HIV test” were significantly positively associated with the intention. The belief “It causes many worries for patients if I offer or counsel them about HIV test” was significantly negatively associated with the intention. The intention was also negatively associated with the beliefs: “Patients are at low risk of HIV and do not need offering or counseling on HIV test” and “If I offered or counseled patients about HIV test, I would spend more time.” The intention was not associated with the beliefs “If I offer or counsel patients about HIV test, I would feel discomforted” and “Patients think that I should offer or counsel them about HIV tests.”

The relatively high relevant beliefs regarding intention are:” If I offer or counsel patients on HIV test, I feel that I am doing something positive”; “My manager thinks that I should offer or counsel patients about HIV test”; “Patients are at low risk of HIV and do not need offering or counseling on HIV test” and “If I offered or counseled patients about HIV test, I would spend more time.” This is because of their associations with intention and the distribution of their scores. The mean score of the belief “If I offered or counseled patients about HIV test, I would spend more time with them” was above the middle of the scale, indicating that a considerable proportion of the participant had this belief. The belief “If I offer or counsel patients about HIV test, I would feel discomforted” is not relevant because it is not associated with the intention.

## Discussion

This study's objective was to assess the psychosocial beliefs associated with HCPs' intention and behavior to offer HTC services and establish the relevance of these beliefs for interventions to promote HTC provision-related intention and behavior among HCPs. Our study results suggest a variation in the association of psychosocial beliefs with the intention and behavior of HCPs to offer HTC services. They also vary in relevance to interventions to promote the intention and behavior of HCPs to offer HTC services.

The study findings showed a strong negative association between the belief “It causes many worries for patients if I offer or counsel them about HIV test” with intention and behavior to offer HTC services. The mean scores of the belief were above the middle of the scale for intention and behavior (not offering HTC services), indicating that more than half of the participants believed that providing HTC services increases their patients' worries. This could be explained by the high stigma surrounding HIV infection in Sudan (33). The combination of the above mid-scale belief scores and the significant negative association highlights the potential adverse effect of this belief on HCPs' intention and behavior to offer HTC services and views it as a highly relevant belief for intervention to promote intention and behavior to the provision of HTC services. This finding agrees with a systematic review of the implementation of provider-initiated testing and counseling in Sub-Saharan Africa that reported health care staff might not offer HIV tests because of a fear of offending patients (6). It is also in accordance with a study of general practitioners in primary care in Belgium which suggested fear of offending patients as a barrier to offering HIV testing and counseling services (34). Additionally, a study of HCPs found that HCPs' concern about offending patients was negatively associated with offering HIV tests (35). This relation is well-established and implies a need for an intervention message that helps providers to provide HTC services without offending their patients.

Our analysis also shows that the belief “My manager thinks I should offer or counsel patients about HIV test” has a significant positive association with intention and behavior to offer HTC services. This belief, with mean scores around the middle of the scale, suggests that about half of the participants were already not convinced that managers supported the provision of HTC services. This distribution of means scores and positive association with behavior and intention makes it a relatively highly relevant belief for intervention. The influence of managers on health workers' behavior was confirmed in a study from South Africa, which reported a significant association between the managers' and healthcare providers' practices (36). Another study also pointed out that perception of management practices and support is essential for patient care delivery and expansion of HIV testing (37). Therefore, a lack of management support can be a barrier to offering HTC services. This might highlight the strong influence of normative beliefs on HCPs' intentions and behaviors. A study of health care professionals investigating the intention to use clinical guidelines showed an association between providers' intentions and subjective norms (38). Reinforcing Sudanese HCPs' belief that managers support offering HTC services can improve their intentions and behaviors of providing HTC services.

Furthermore, our study found that HCPs' perception that patients are at low risk of HIV and do not need offering or counseling on HIV test is negatively associated with the intention and behavior to offer HTC services. This belief is a relevant high belief due to negative associations and the distribution of its mean scores around the middle of the scale, indicating that many participants had this belief which prevented them from offering HTC services. This perception could be explained by the low prevalence of HIV infection in Sudan (29). The low prevalent cases of HIV infection limited the contact of HCPs with HIV patients, reinforcing this belief among them and lowering their readiness to offer HIV tests to patients. The absence of HIV test suggestions by health care providers based on patient risk will result in missing opportunities for detecting patients infected with HIV (39). Also, the study established that the belief “If I offered or counseled patients about HIV test, I would spend more time with them” is negatively associated with the intention and behavior of HCPs to offer HTC services. The participants' response scores for intention and not performing HTC services behavior are above the middle of the scale, indicating that more than half of the participants are convinced that providing HTC services takes more time. This is in line with a study conducted among healthcare providers, which found that providers might perceive a lack of time as a barrier to offering HIV tests (40). 'This perception is possible because of the high workload and time constraints healthcare providers face in their work (15). This finding highlights the need for intervention that reduces time constraint perceptions among health care providers to promote the offering of HTC services (41).

Furthermore, the study found that the belief “My colleagues think I should offer or counsel patients about HIV test” is weakly associated with intention and behavior to offer HTC services. The mean score of this belief is in the middle of the scale, indicating that many participants disagree that their colleagues approve of offering HTC to patients. However, it is a relatively low relevant belief for intervention because of the weak association with the intention and behavior.

The results of this study should be interpreted in the context of some limitations. This study was conducted among HCPs in Kassala State. The cross-sectional study design limits its ability to establish the causal effect of psychosocial beliefs on the intention and behavior of HCPs. Further, a follow-up study is needed to measure beliefs related to HCPs' intention and behavior of offering HTC services over time. We assessed participants through a self-administered questionnaire. That may be subjected to some social desirability bias. Participants were invited to complete in a private room and assured of responses' anonymity and confidentiality to control the social desirability bias on their responses. Another limitation may be conducting the study in one state in Sudan, which may affect the generalizability of the study result to healthcare providers in the other states. Because healthcare providers in Sudan share similar characteristics and beliefs, we believe that our study findings can be used among other healthcare providers in Sudan and other similar settings.

## Conclusion

Psychosocial beliefs influence HCPs' intention and behavior to offer HTC services. This study showed that psychosocial beliefs vary in association with the intention and behavior of HCPs to offer HTC services to patients. Also, they differ in relevance to be targeted with interventions. The belief “My manager thinks I should offer or counsel patients about HIV test” was positively associated with HCPs' intention and behavior to offer HTC services and a relevant high belief for targeting by interventions. The beliefs “It causes many worries for patients if I offer or counsel them about HIV test” and “If I offered or counseled patients about HIV test, I would spend more time with them” were negatively associated with intention and behavior. They were highly relevant beliefs for interventions. Also, the belief “Patients are at low risk of HIV and do not need offering or counseling about HIV test” was negatively related to intention and behavior to offer HTC services and was a highly relevant candidate for intervention selection. However, the belief “My colleagues think I should offer or counsel patients about HIV test” was weakly associated with intention and behavior and was a low relevant belief for intervention. Interventions to promote intention and behavior to offer HTC services among health care providers to be more effective should be based on targeting the most relevant beliefs.

## Data availability statement

The raw data supporting the conclusions of this article will be made available by the authors, without undue reservation.

## Author contributions

AI collected the data. AI, RC, and HV contributed to the analysis, drafted the content of the study, and revised and provided approval for publication of the Study. All authors contributed to framing the study.

## Conflict of interest

The authors declare that the research was conducted in the absence of any commercial or financial relationships that could be construed as a potential conflict of interest.

## Publisher's note

All claims expressed in this article are solely those of the authors and do not necessarily represent those of their affiliated organizations, or those of the publisher, the editors and the reviewers. Any product that may be evaluated in this article, or claim that may be made by its manufacturer, is not guaranteed or endorsed by the publisher.
